# m^6^A Regulator-Mediated Methylation Modification Patterns and Characteristics of Immunity in Blood Leukocytes of COVID-19 Patients

**DOI:** 10.3389/fimmu.2021.774776

**Published:** 2021-11-30

**Authors:** Xiangmin Qiu, Xiaoliang Hua, Qianyin Li, Qin Zhou, Juan Chen

**Affiliations:** ^1^ The Ministry of Education Key Laboratory of Laboratory Medical Diagnostics, The College of Laboratory Medicine, Chongqing Medical University, Chongqing, China; ^2^ Department of Urology, The First Affiliated Hospital of Anhui Medical University, Hefei, China; ^3^ Anhui Province Key Laboratory of Genitourinary Diseases, Anhui Medical University, Hefei, China

**Keywords:** COVID-19, immune characteristics, m^6^A methylation modification, protective model, leukocytes

## Abstract

Both RNA N6-methyladenosine (m^6^A) modification of SARS-CoV-2 and immune characteristics of the human body have been reported to play an important role in COVID-19, but how the m^6^A methylation modification of leukocytes responds to the virus infection remains unknown. Based on the RNA-seq of 126 samples from the GEO database, we disclosed that there is a remarkably higher m^6^A modification level of blood leukocytes in patients with COVID-19 compared to patients without COVID-19, and this difference was related to CD4^+^ T cells. Two clusters were identified by unsupervised clustering, m^6^A cluster A characterized by T cell activation had a higher prognosis than m^6^A cluster B. Elevated metabolism level, blockage of the immune checkpoint, and lower level of m6A score were observed in m^6^A cluster B. A protective model was constructed based on nine selected genes and it exhibited an excellent predictive value in COVID-19. Further analysis revealed that the protective score was positively correlated to HFD45 and ventilator-free days, while negatively correlated to SOFA score, APACHE-II score, and crp. Our works systematically depicted a complicated correlation between m^6^A methylation modification and host lymphocytes in patients infected with SARS-CoV-2 and provided a well-performing model to predict the patients’ outcomes.

## Introduction

Recently, a total of seven internal modifications have been discovered on mRNA: N^1^-methyladenosine (m^1^A), N^4^-acetylcytidine (ac^4^C), 5-methylcytidine (m^5^C), N6-methyladenosine (m^6^A), N^7^-methylguanosine (m^7^G), ribose methylations (N_m_), and pseudouridine (Ψ) (1). mRNA modification is a reversible process mediated by “writers,” “readers,” and “erasers”, and m^6^A, which was first reported by Desrosiers in 1974, is the most common type of mRNA modification (2). mRNA can be methylated by the writers (*METTL3* and *METTL14*), and translated into protein efficiently with the help of the readers (*YTHDF1* and *YTHDF2*), while the erasers (*FTO* and *ALKBH5*) demethylate the residues (3–7). On the molecular level, m^6^A can affect RNA structures, influence the accessibility of RNA-binding motifs to their RNA-binding proteins, promote the initiation of miRNA biogenesis, and facilitate the translation of proteins (8). With respect to biological function, m^6^A has been shown to affect individual development, infertility, carcinogenesis, stemness, meiosis, circadian rhythm, and control various aspects of immunity, including immune recognition, activation of innate and adaptive immune responses, and cell fate decisions (9, 10). For instance, deletion of *YTHDF2* delays mouse neuronal development through impaired proliferation and differentiation of neural stem and progenitor cells (11). In addition, the function of m^6^A can be induced by environmental stimuli or cellular signaling pathways. When monkey kidney cells were infected with enterovirus type 71, *YTHDF1* and *YTHDF2* were upregulated and distributed into both the cytosol and the nucleus (12).

Patients infected with severe acute respiratory syndrome coronavirus clade 2 (SARS-CoV-2) exhibited various changes in the immune system such as those on immune cell fractions, the expression level of the immune checkpoint, cytokine storm, and so on. During the early stages of COVID-19 infection, lymphocyte fractions might change, for example, the numbers of CD4^+^ and CD8^+^ T cells are significantly elevated due to immune defense against the virus (13). Another report noted that mild cases of COVID-19 had a greater proportion of CD8^+^ T cells than CD4^+^ T cells (14). Apart from the activated T cells, antibody responses in the extrafollicular zone were also stimulated to protect the organism against SARS-CoV-2 invasion (15). Moreover, some immune function assays were also conducted on macaques infected with SARS-CoV-2, and researchers obtained significant results such as a delayed immune response, increased inflammatory cytokine storm, and declined T cell function during the infections (16).

Recent studies have unveiled the alteration of m^6^A modification in host cells and SARS-CoV-2. Li et al. noted that *METTL3* and *METTL14* gene expression in lung tissues was significantly downregulated, whereas the expression levels of most of the inflammatory genes and insulin stimulated genes (ISGs) were increased in COVID-19 patients than in healthy individuals. The SARS-CoV-2 virus utilizes host *METTL3* to modify viral RNA and to evade host cell immune responses (17). SARS-CoV-2 infections were also found to trigger m^6^A modification machineries re-localization and enhance the abundance of m^6^A in Vero and Huh7 cells (18). Although these findings provide evidence of the m^6^A methylome interaction between host cells and SARS-CoV-2, current studies focused primarily on a few m^6^A-related genes and nearly all were performed using model cells such as Caco2 and Huh7, which may not adequately reflect the actual situation of m^6^A methylome modifications in immune cells in patients with SARS-CoV-2 infection. Consequently, there is an urgent need to explore the m^6^A methylome modification profile in immune cells and the cross-talk between m^6^A modification and immune functions. Our aim is to explore whether there is a discrepancy in the expression levels of m^6^A regulators between patients with and without COVID-19, and how m^6^A methylome modification affects the immune function of lymphocytes.

In this study, we systematically depicted the immune profiles in patients with and without COVID-19 and the correlation between m^6^A and lymphocytes between these groups. Based on the expression levels of 20 m^6^A regulators, we discovered two distinctive m^6^A modification patterns in blood lymphocytes of COVID-19 patients. Surprisingly, there were differences in metabolism, immune cell compositions, and immune checkpoints between the two groups of patients. To better quantify the m^6^A modification level in each patient group, we established a scoring system termed the m^6^A score. This system was further analyzed between two m^6^A patterns and different clinical manifestation groups. Finally, we generated a protective model to accurately predict the clinical outcomes of patients and to determine the presence of SARS-CoV-2 infection among patients.

## Materials and Methods

### Processing of Data Obtained From a GEO Dataset

RNA-seq data of 126 samples, including those of 100 patients with COVID-19 and 26 patients without COVID-19 were obtained from a GEO dataset (GSE157103) (19). Clinical information obtained included age, diabetic status, ICU status, and hospital-free days at day 45 (HFD45). The HFD45 assigns a zero value (0-free days) to patients who remained admitted for over 45 days or to those who died while they were admitted, and higher HFD45 values are assigned to patients with shorter hospitalization times and milder disease severity.

### GSVA Analysis and Functional Annotation

To estimate the biological function between different m^6^A clusters or between patients with or without COVID-19, we conducted GSVA enrichment analysis using the “GSVA” R package, which estimates the variations of pathway activity over a sample population in an unsupervised manner (20). The “h.all.symbols” and “c5.go.bp.symbols” were downloaded from the MSigDB database for GSVA analysis. The significantly enriched pathways were filtered by an adjusted P value of <0.05. To investigate the potential biological functions of DEGs of two m^6^A clusters and of individuals with or without COVID-19, the “clusterProfiler” package in R was used to perform enrichment analysis (21).

### Estimation of Immune Cell Infiltration Fractions

The abundance of immune cells was determined by cell type identification by “CIBERSORT”, an algorithm that combines support vector regression from purified leukocyte subsets (https://cibersort.stanford.edu/). The LM22 signature gene matrix served as an input of the “CIBERSORT” algorithm to analyze the RNA-seq data of 126 samples, and all samples with a P value of <0.05 were included (22).

### Generation of m^6^A Score

To quantify the m^6^A modification level per individual, we established an evaluation index called the m^6^A score.

1) Acquisition of significant DEGs. TPM data were log2-transformed, and the DEGs were acquired from the two m^6^A clusters using the “limma” package. We used HFD45 = 26 as the cutoff value and categorized COVID-19 patients into two groups. Each gene with differential expression between the two groups was analyzed by the *t-*test. The significant DEGs were extracted for further analysis.
2) Construction of the m^6^A score. A PCA analysis was adopted to focus on the well-correlated genes in the set. PC1 and PC2 were extracted to form signature scores. Later, we applied a method similar to GGI to construct the m^6^A score (23).

$${\text{m}}^{6}\text{A score}=\Sigma (\text{PC}1_{\text{i}}+\text{PC}2_{\text{i}})$$

### Unsupervised Clustering of COVID-19 Patients

A total of 20 m^6^A genes were obtained from the GEO dataset, including eleven readers (*YTHDC1*, *YTHDF2*, *YTHDF1*, *ELAVL1*, *YTHDC2*, *FMR1*, *HNRNPA2B1*, *IGF2BP1*, *LRPPRC*, *YTHDF3*, and *HNRNPC*), seven writers (*ZC3H13*, *RBM15B*, *RBM15*, *CBLL1*, *WTAP*, *METTL14*, and *METTL3*), and two erasers (*ALKBH5* and *FTO*). An unsupervised clustering algorithm performed by the “ConsensusClusterPlus” package was used on the basis of the m^6^A genes to classify COVID-19 patients into different subtypes (24).

### Construction of the Protective Model

Comparison of the two groups yielded a total of 4,565 genes with differential expression. We constructed the LASSO model in the patient’s cohort on the basis of these DEGs by using the “glmnet” package. The final signatures were filtered by determining the appropriate λ value with 20-fold cross-validation and “deviance” as the target parameter. The coefficients of the final signatures were used to calculate the protective score as follows: protective score = ∑_i_ Coefficients_i_ × Expression level of signature_i_. The patients were divided into two clusters: the training cohort consisted of 70% of the patients while the validation cohort consisted of 30% of the patients. The model constructed in the training cohort was validated in the validation cohort. Receiver operating characteristic (ROC) curves were plotted with AUC scores using the R package “plotROC” to evaluate the performance of the model.

### Statistical Analysis

Differences between the two groups were compared using the Wilcoxon sum-rank test and the *t*-test. The protective score, HFD45, SOFA score, APACHE-II score, crp, and ventilator-free days were subjected to correlation analysis using the Pearson correlation test with the “pancor” package (https://github.com/xuzhougeng/pancor/tree/master/R). All statistical tests conducted were two-sided, and a p value of <0.05 was considered statistically significant.

## Results

### Upregulation of m^6^A Regulators and Activation of the Immune System in COVID-19 Patients

**Figure 1:**
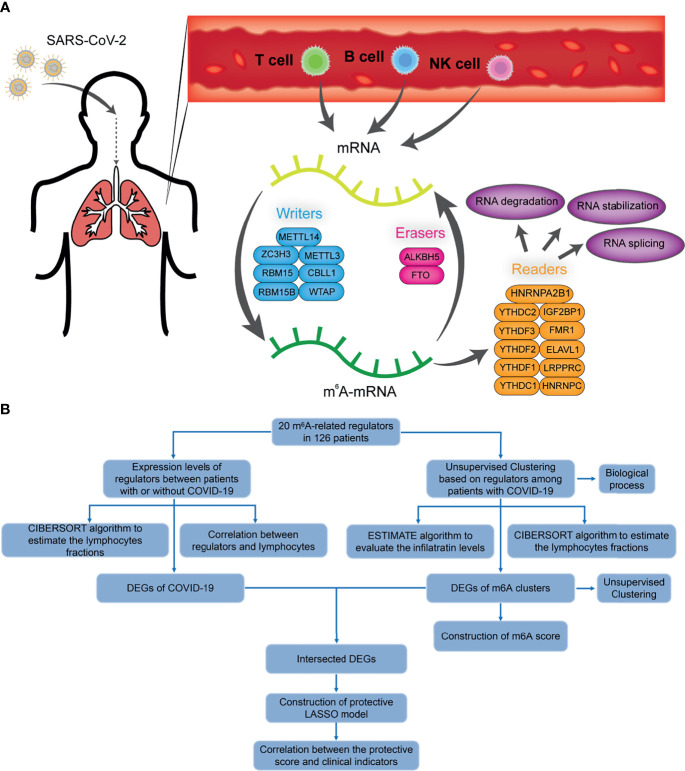
The diagram and workflow of the project. **(A)** The overview of m^6^A RNA methylation modification in blood lymphocytes of patients infected with SARS-CoV-2, including ‘writers’, ‘readers’, and ‘erasers’. **(B)** The study flow chart.

**Figure 2:**
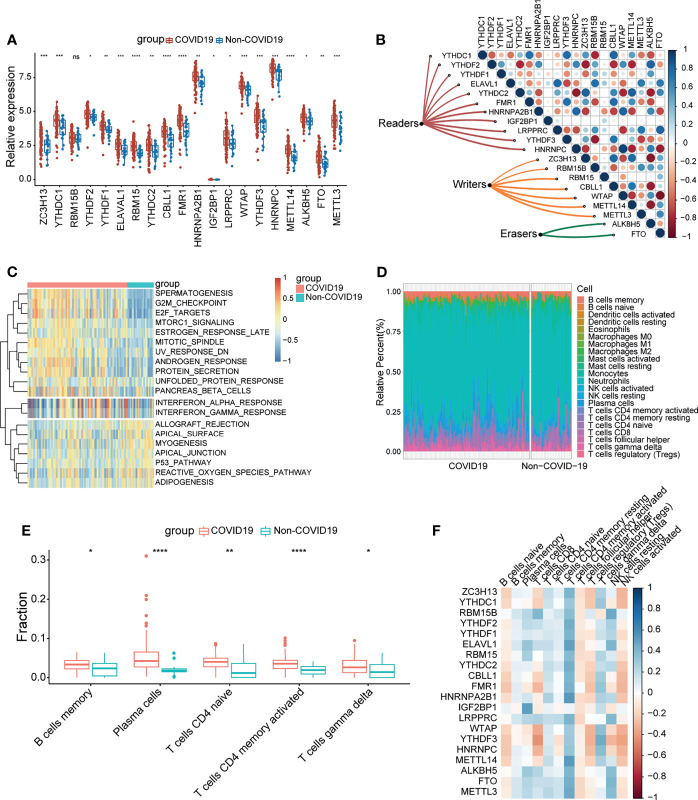
COVID-19 patients were characterized by upregulated m^6^A genes and activation of the lymphocytes. **(A)** The expression of 20 m^6^A genes of blood leukocytes between patients with or without COVID-19. **(B)** Correlation plot of 20 m^6^A genes. The positive correlation was marked with blue, and negative correlation was marked with red. The size of circle represents the absolute value of correlation coefficients. **(C)** GSVA enrichment analysis showing activated interferon pathways in COVID-19 patients. Red represents high expression, blue represents low expression. **(D)** The abundance of leukocytes in patients with or without COVID-19. **(E)** The significant leukocytes fractions in patients with or without COVID-19. **(F)** The heatmap of correlation between leukocytes and m^6^A genes. The positive correlation was marked with blue, and negative correlation was marked with red. *p < 0.05, **p < 0.01, ***p < 0.001, ****p < 0.0001, ns, no significance.

A sketch map was depicted to reflect the m^6^A modification of blood lymphocytes of patients infected with SARS-CoV-2 (**Figure 1A**). The gene expression profiles and corresponding clinical data of patients with or without COVID-19 were downloaded from the Gene Expression Omnibus (GEO) database for subsequent analyses. **Figure 1B** shows the workflow. We curated and analyzed a set of 20 acknowledged m^6^A regulators (11 writers, 7 readers, and 2 erasers) to identify distinct m^6^A methylation modification patterns. Expression profiling of blood leukocytes revealed that the expression levels of all m^6^A regulators were significantly upregulated in patients with COVID-19 (P <0.05) (**Figure 2A**). To explore the association between different m^6^A regulators, we depicted the correlation patterns between three types of m^6^A regulators (**Figure 2B**). Surprisingly, m^6^A regulators of the same type, such as YTHDF2 and YTHDC1, show strong antagonistic action (coefficient = −0.6). Simultaneously, m^6^A regulators from different types, such as HNRNPC and WTAP, can also exhibit synergistic effects (coefficient = 0.94). We further analyzed the relevance of the co-expression of regulators and found a significant correlation between YTHDF2 and other regulators, with the highest correlation coefficient between YTHDF2 and ALKBH5 (coefficient = 0.82). Of course, these are predicted interactions that provide a theoretical basis for later experimental validation. The above results provide evidence to the regulatory balance among the 20 m^6^A regulators.

To determine whether there are alterations in the immune system between the COVID-19 and non-COVID-19 patient groups, gene set variation analysis (GSVA) was conducted to show a difference in well-defined biological states or processes between patients with or without COVID-19, indicating that interferon responses were remarkably upregulated in COVID-19 patients (**Figure 2C**). We simultaneously analyzed the fraction of 22 immune cell types between the two groups based on the CIBERSORT algorithm (**Figure 2D**), and the results revealed that COVID-19 patients had higher infiltration levels of memory B cells, plasma cells, naïve CD4 T cells, activated CD4 memory T cells, and gamma delta T cells (**Figure 2E**). These findings suggested that SARS-CoV-2 infection remarkably activates the immune system. Moreover, correlation analysis underlined that activated CD4 memory T cells were positively correlated with m^6^A regulators (**Figure 2F**). Combined with the above results, it can be inferred that the high level of activated CD4 memory T cells in COVID-19 patients may be due to the elevated expression level of m^6^A regulators. The above results suggested that m^6^A regulators may play a pivotal role in the molecular traits and immune infiltration phenotype in COVID-19 patients.

### Patterns of m^6^A Regulators and Biological Function of Each Pattern

**Figure 3:**
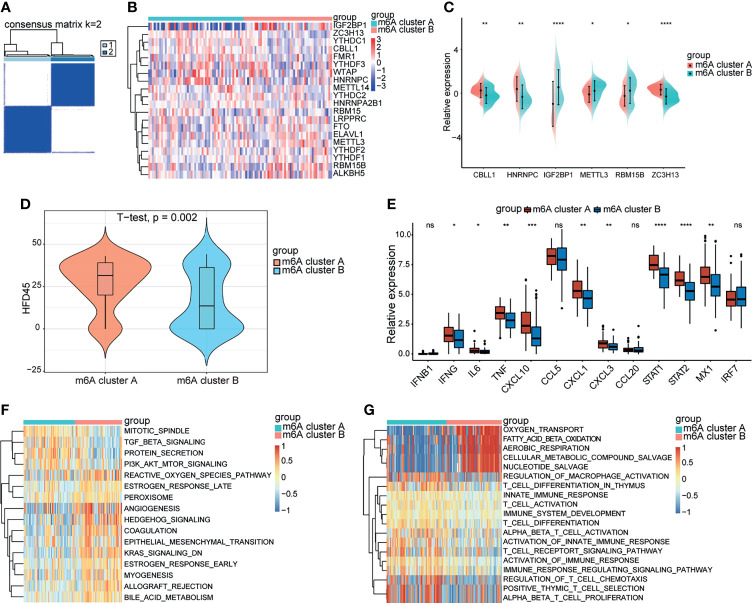
Biological progression between the two m^6^A clusters. **(A)** Consensus clustering matrix for k = 2. **(B)** The heatmap of m^6^A genes between the two m^6^A clusters. Red represents high expression, blue represents low expression. **(C)** Expression levels of significant m^6^A genes between the two m^6^A clusters. **(D)** The HFD45 between the two m^6^A clusters. **(E)** The innate immune pathways-related genes between the m^6^A clusters. **(F, G)** GSVA analysis showing the activation of classical pathways and distinct biological processes in metabolism and immune response. *p < 0.05, **p < 0.01, ***p < 0.001, ****p < 0.0001, ns, no significance.

A consistent unsupervised methodology was employed to obtain a clustering result for subsequent analysis. The consensus matrix showed that the unsupervised algorithm based on the 20 regulators could clearly distinguish the samples, and each sample in a cluster possessed a high correlation (**Figures 3A**, **S1A–C**). The consensus distributions and delta area for k (2–5) are displayed in the empirical cumulative distribution function (CDF) plots (**Figures S1D–E**). Given the consensus matrix for the analysis, k = 2 seemed to be the most suitable choice. Accordingly, in this study we clustered COVID-19 patients into two groups, and the principal component analysis (PCA) revealed that the two groups were distinguished clearly (**Figure S1F**). Moreover, compared to the expression levels of m^6^A regulators, a unique m^6^A transcriptional profile was generated between the two m^6^A patterns (**Figure 3B**). m^6^A cluster A showed high expression levels of CBLL1, HNRNPC, and ZC3H13, while m^6^A cluster B was characterized by elevated expression of IGF2BP1, METTL3, and RBM15B (**Figure 3C**). METTL3, which was previously reported by Hu, was considered to be an important part of the methyltransferase complex (5), suggesting that the m^6^A cluster B might have a higher level of m^6^A methylation modification in lymphocytes compared to m^6^A cluster A. Some host proviral genes that are essential for the survival of SARS-CoV-2 have been reported (25–31). We examined the expression levels of these genes in the two clusters. As shown in the result (**Figures S2A–C**), proviral genes were significantly upregulated in m^6^A cluster A relative to m^6^A cluster B. The hospital-free day 45 (HFD45) between the two clusters was compared, and the results revealed a better prognosis for m^6^A cluster A (**Figure 3D**). Thus, we speculated that the upregulated expression of proviral genes might be associated with a low level of m^6^A methylation modification in lymphocytes, leading to better outcomes in COVID-19 patients. We subsequently explored effectors downstream of the innate immune pathways between the two groups, and the results showed that IFN genes and IFN-stimulated genes were significantly upregulated in m^6^A cluster A (**Figure 3E**), implying that lymphocytes of this cluster were significantly stimulated to release antiviral proteins such as IFN.

GVSA analysis was applied to further explore the biological differences between the two groups. The results revealed that KRAS and TGFβ signaling was upregulated in m^6^A cluster A while PI3K-AKT-mTOR signaling was downregulated in m^6^A cluster B (**Figure 3F**). Otherwise, the significant pathways also focused on metabolism and immune system activation. m^6^A cluster B was remarkably related to oxygen transport, fatty acid β-oxidation, aerobic respiration, cellular metabolism compound salvage, and nucleotide salvage. T cell pathways, such as T cell activation, T cell differentiation, T cell chemotaxis, and T cell proliferation, were significantly enriched in m^6^A cluster A (**Figure 3G**). Thus, we hypothesized that m^6^A cluster A might be involved in various processes in T cells, such as development and function.

### Immune Infiltration and Immune Checkpoint Characteristics in m^6^A Patterns

**Figure 4:**
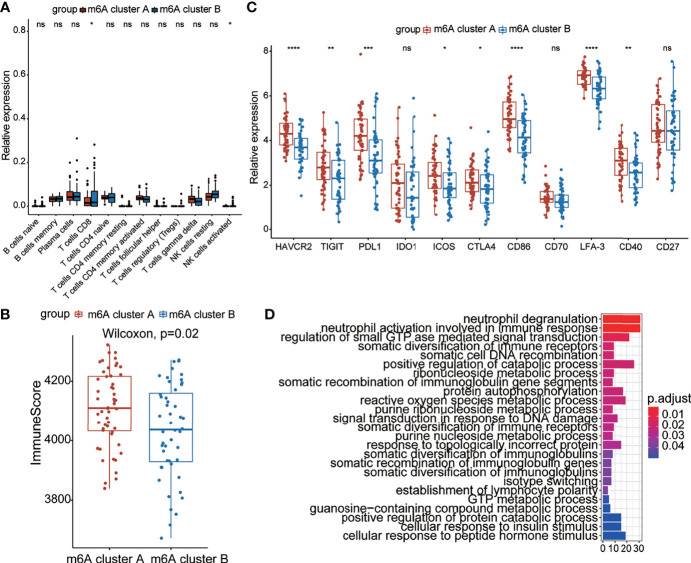
Immune characteristics between the two m^6^A clusters. **(A)** The abundance of leukocytes between the m^6^A clusters. **(B)** The immunoscore between the two m^6^A clusters. **(C)** Expression levels of immune checkpoint genes between the m^6^A clusters. **(D)** The KEGG enrichment analysis based on DEGs of the two clusters. The color bar represents the p values of the pathways. *p < 0.05, **p < 0.01, ***p < 0.001, ****p < 0.0001, ns, no significance.

Recent studies have shown that m^6^A modification of RNA plays an essential role in the formation of immune responses and the immune environment. In order to further define the role of m^6^A modification patterns in the immune environment, we compared the components of different lymphocytes between two m^6^A clusters by using the CIBERSORT method (**Figure 4A**). We found that m^6^A cluster A had higher expression of CD8^+^ T cells and activated NK cells than m^6^A cluster B, which is consistent with the above results. To better illustrate the level of infiltration in the two clusters, we leveraged the ESTIMATE algorithm to evaluate the infiltration level of immune cells. The results revealed that m^6^A cluster A exhibited a high immune score, which suggested that m6A cluster A had prominently elevated infiltration of immune cells (**Figure 4B**). These results illustrated the differences in immune infiltration between the two modification patterns.

We further analyzed the expression of typical immune-related genes and immune checkpoint-related genes in the groups with different modification patterns. The results uncovered that stimulator, inhibitor, and MHC-related genes were remarkably elevated in m6A cluster A than in m6A cluster B (**Figures S3A–C**), suggesting that m6A cluster A had a higher immune response than m^6^A cluster B. Interestingly, m^6^A cluster A could be remarkably distinguished from m^6^A cluster B in the immune checkpoint. In particular, we found that the expression of checkpoint inhibitor-related genes, such as *HAVCR2*, *TIGIT*, *PD*-*L1*, *ICOS*, *CTLA4*, *CD86*, *LFA*-3, and *CD40*, in the m^6^A cluster A was prominently higher than that in m^6^A cluster B, which meant that the former cluster might benefit from immune therapy (**Figure 4C**). To better illustrate the biological behaviors between the two groups, Kyoto Encyclopedia of Genes and Genomes (KEGG) enrichment was performed using the “clusterProfiler” package. Surprisingly, immunity- and metabolism-related genes were primarily enriched (**Figure 4D**), which is the same as the biological process between patients with and without COVID-19. Based on the above results, it could be said that there were distinct immune infiltration and immune checkpoint characteristics between the two groups with different modification patterns.

### Construction of m^6^A Signatures

**Figure 5:**
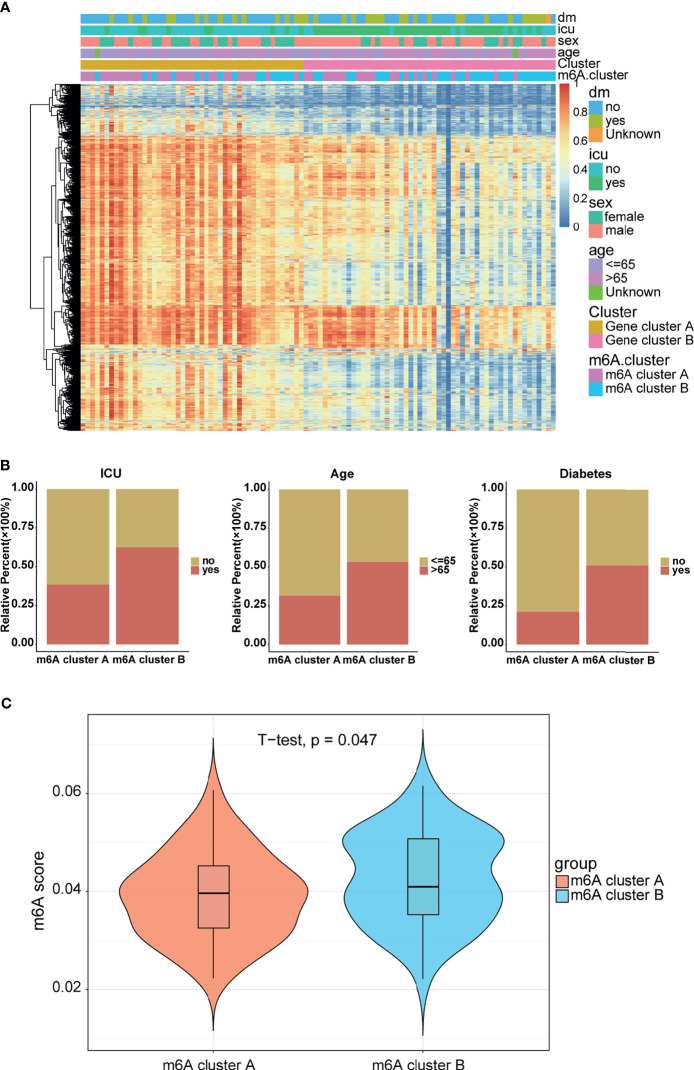
Clinical manifestations and m^6^A modification levels between the two m^6^A clusters. **(A)** Heatmap of the DEGs between the gene clusters. m^6^A cluster and clinical feature annotation was used. **(B)** ICU, age, and diabetes proportions between the m^6^A clusters. **(C)** m6A score between the m^6^A clusters.

To further verify the reasonability of classification based on m^6^A-related genes, we first analyzed the differentially expressed genes (DEGs) using the “limma” package (32). DEGs were identified with cutoff criteria of |logFC| >1 and P <0.05, and finally we screened 6,771 DEGs. Subsequently, unsupervised consensus clustering analysis was conducted on the basis of the DEGs using the R package “ConsensusClusterPlus” to categorize the patients into different genomic subtypes. The delta area and consensus distributions for k (2–5) are displayed in the empirical CDF plots (**Figures S4A–E**). Consistent with the classification of m^6^A modification patterns, the unsupervised algorithm clustered two unique genomic subtypes. We designated these subtypes as “Gene cluster A” and “Gene cluster B”, and this classification was further confirmed by PCA (**Figure S4F**). Coincidently, there were more m^6^A-related genes in Gene cluster A than in Gene cluster B (**Figure S5G**), although there were no significant differences in the HFD45 score (**Figure S5A**). These analyses indicated that the two m^6^A modification models existed in COVID-19 patients and that the classification based on m^6^A-related genes was reasonable and could be explained. Furthermore, we analyzed the overall expression of the DEGs, and the results are depicted in a heatmap (**Figure 5A**), which illustrates the existence of a distinct genomic expression profile between the two groups. Later, we observed the proportions of clinical manifestations of the COVID-19 patients between the two m^6^A clusters (**Figure 5B**). It indicated that the patients in m^6^A cluster B were more likely to be admitted to the ICU or have diabetes than patients in m^6^A cluster A. Concomitantly, patients in Gene cluster A were characterized by an age of <65 years.

Considering the unique heterogeneity of m^6^A modification patterns, we defined an indicator to establish a scoring system to comprehensively quantify the m^6^A modification pattern of patients with COVID-19, which is termed as the m^6^A score. Further analysis revealed a lower m^6^A score in m^6^A cluster A than in m^6^A cluster B (**Figure 5C**). Combined with the conclusion that the m^6^A cluster A had a higher HFD45 than m^6^A cluster B, it can be inferred that the m^6^A score was associated with poor survival. However, there was no significant difference in the m^6^A signature between Gene clusters A and B (**Figure S5B**). Similar results were discovered between different clinical groups (**Figures S5C–F**). To better illustrate the potential function of the m^6^A score, we analyzed the correlation between the m^6^A score and common pathways. Based on the results of the correlation analysis, the m^6^A score was mainly positively correlated with glycerolipid metabolism and autoimmune thyroid disease, and negatively correlated with the regulation of autophagy, peroxisome, drug metabolism, glycerophospholipid metabolism, and RNA degradation (**Figure S5G**). These results demonstrated that the m^6^A score might be closely related to metabolic pathways.

### Construction and Validation of an m^6^A-Related Protective Model

**Figure 6:**
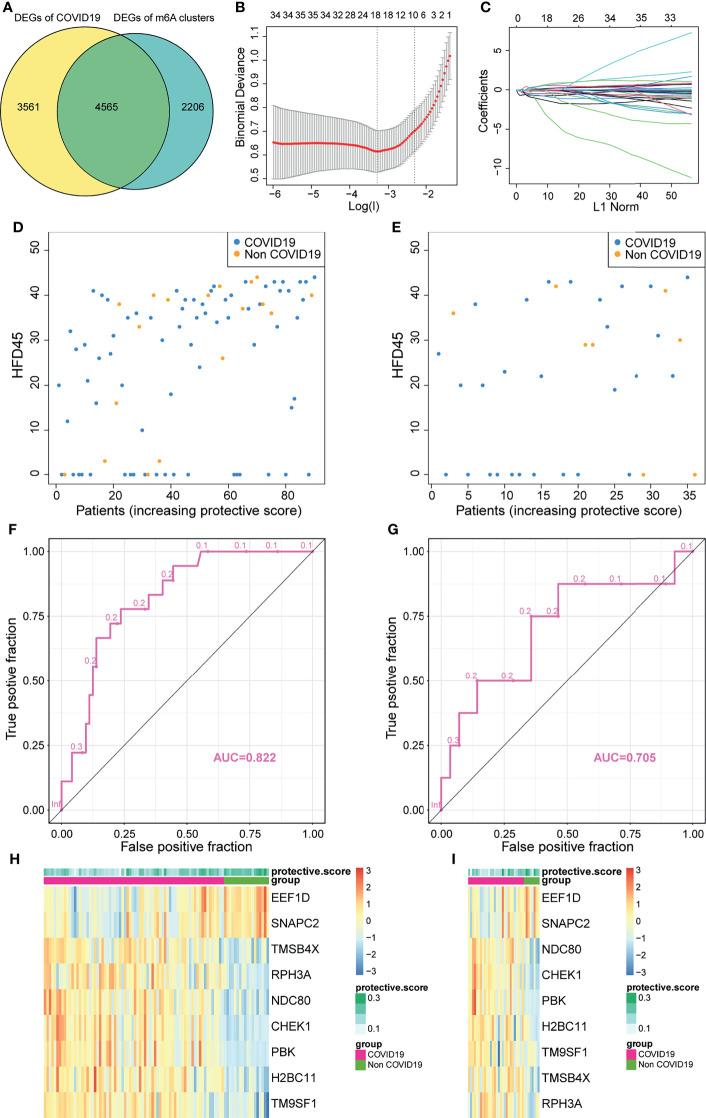
Construction of a protective model to predict patients with COVID-19. **(A)** Venn plot between DEGs of COVID-19 and DEGs of clusters. **(B, C)** Construction of a protective model based on intersecting DEGs. **(D, E)** The HFD45 of patients in the training set and testing set ranked by protective score. **(F, G)** AUC of patients in the training set and testing set. **(H, I)** The heatmap of the model genes in the training set and testing set.

In view of the necessity to detect COVID-19 in individuals and the importance of m^6^A regulators, an accurate predictive model needs to be built. We analyzed the intersections between DEGs of two m^6^A clusters and DEGs of COVID-19 and COVID-19 individuals, and acquired a total of 4,565 overlapped DEGs (**Figure 6A**). These DEGs were regarded as candidate genes for least absolute shrinkage and selection operator (LASSO) regression analysis based on the least square method. In the cross-validation process, lambda-min was regarded as the optimal value (**Figure 6B**). **Figure 6C** presents the calculated regression coefficient. Finally, nine model-related genes were obtained, which were then used to construct a protective model. The against-COVID-19 signature was as follows: protective score = (−0.40363 × CHEK1) + (−0.00647 × NDC80) + (−0.03129 × PBK) + (−0.27285 × H2BC11) + (−0.06532 × TMSB4X) + (−0.04487 × RPH3A) + (−0.19111 × EEF1D) + (0.083252 × SNAPC2). Further analysis demonstrated that both in the training and validation sets, patients with high protective scores had a higher level of HFD45 and were more likely to protect themselves against COVID-19 infections than those with low protective scores (**Figures 6D** **, E**). Moreover, the area under the ROC curve (AUC) values of the model in the training and validation sets were 0.822 and 0.705, respectively (**Figures 6F** **, G**), suggesting the excellent performance of the protective model. The heatmaps of the model-related genes were plotted, which indicated a distinct difference in expression levels between the patients with and without COVID-19 in both sets (**Figures 6H** **, I**).

**Figure 7:**
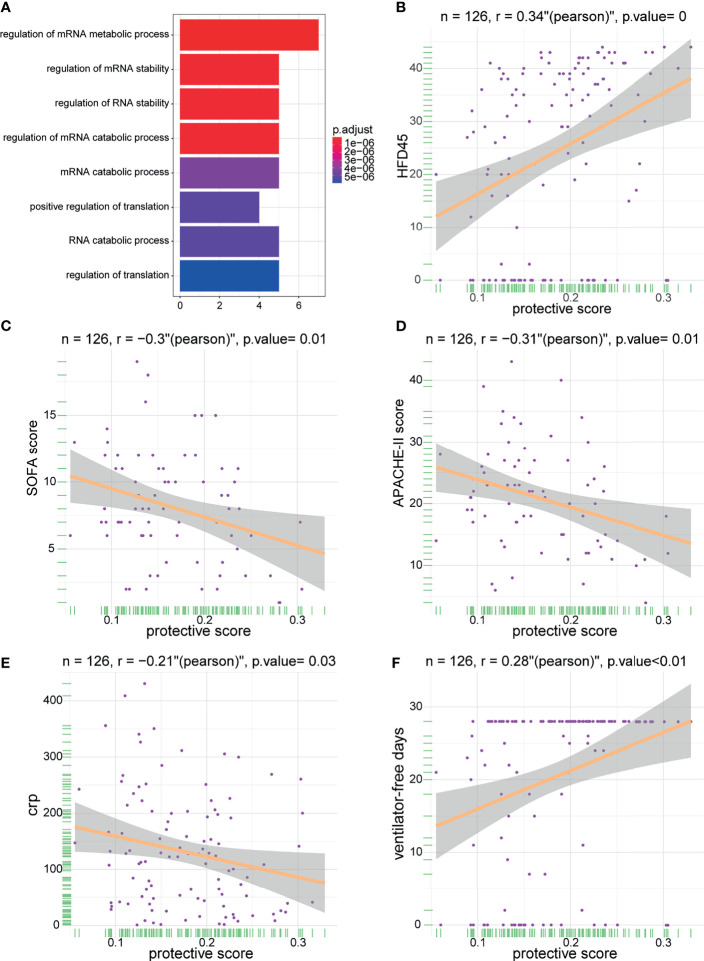
The enrichment of model genes and correlations between protective score and clinical information. **(A)** The biological process of the model-related genes. **(B–F)** The correlations between protective score and HFD45 **(B)**, SOFA score **(C)**, APACHE-II score **(D)**, crp **(E),** and ventilator-free days **(F)**.

To delineate the role and potential mechanisms of the predictive performance of the model, we conducted gene ontology (GO) and KEGG analyses of model-related genes. The results of the analyses revealed that the model was mainly related to external factors, cell cycle, and viral carcinogenesis (**Figure 7A**). These findings indicated that the protective model can precisely predict the probability of patients infected with SARS-CoV-2. Later, we studied the correlation between the protective score of the model and clinical information (**Figures 7B** **–F**), which illustrated that a high protective score was positively correlated with HFD45 and ventilator-free days, whereas a high protective score was negatively correlated with SOFA score, APACHE-II score, and C-reactive protein (crp). Taken together, our findings demonstrated the outstanding predictive value of the newly developed protective model and the clinical prognostic value of the protective score.

## Discussion

SARS-CoV-2 is responsible for the severe acute respiratory syndrome. Sokal et al. found that memory B cells in patients responded to COVID-19, while Grifoni et al. and Bert et al. demonstrated that COVID-19-specific CD4^+^ and CD8^+^ T cells are generated during the course of COVID-19 disease (13, 15, 33). Interestingly, SARS-CoV-2 spike-reactive CD4^+^ T cells, which focus on C-terminal S epitopes, can be detected both in patients with COVID-19 and in healthy donors (34). Moreover, a robust CD4^+^ T cell response to SARS-CoV-2 spike (S) protein and nucleoprotein (N) can be observed in individuals who have recovered from SARS-CoV-2 infection (14, 35). Although the phenotype of lymphocyte responses to COVID-19 has been unraveled by researchers, the underlying mechanism of lymphocyte activation in this disease remains obscure.

RNA modification is diverse and vital in the activation and differentiation of lymphocytes. m^6^A methylation can control T cell and B cell homeostasis (36, 37). T follicular helper cell differentiation can also be managed by m^6^A mRNA methylation (38). The above studies primarily focused on communication between tumor and lymphocytes, but whether m^6^A mRNA methylation was altered in the lymphocytes of COVID-19 patients and the potential function of m^6^A modification during infection remains unclear. Thus, there is an urgent need to identify the possible mechanisms and promote our understanding of lymphocyte m^6^A modification in COVID-19 patients.

In this study, we systematically analyzed the m^6^A modification landscape in blood lymphocytes of COVID-19 patients. The m^6^A expression level was significantly upregulated in the blood lymphocytes of COVID-19 patients than in those of patients without COVID-19, suggesting that m^6^A modification might play a vital role in the blood lymphocytes of patients with COVID-19. Later, the correlation between m^6^A regulators was calculated to explore the intricate relationship between the regulators in patients infected with SARS-CoV-2 and uninfected individuals. We discovered a negative correlation between m^6^A regulators of the same type, which proved the existence of an m^6^A modification dynamic balance in COVID-19 patients. The lymphocyte fraction was altered between patients with and without COVID-19. COVID-19 patients had higher levels of B cells and CD4^+^ T cells, which were consistent with the findings reported by Goel et al. and Kared et al. (39, 40). Further, to explore the different m^6^A modification patterns in COVID-19 patients, unsupervised cluster analysis of the expression values of m^6^A regulators identified two distinct modification patterns. m^6^A cluster A exhibited T cell activation and differentiation, while m^6^A cluster B was characterized by metabolism-related biological processes such as fatty acid β-oxidation and nucleotide salvage. Essig et al. and Cortez et al. reported that TGF-β signaling and PI3K-AKT signaling are necessary for T cell differentiation (41, 42). Consistent with the above studies, m^6^A cluster A had a higher level of TGF-β signaling and PI3K-AKT-mTOR signaling, which explained the mechanism of T cell activation and differentiation. Together, it would be reasonable and reliable to state that m^6^A cluster A which had activated T cell function to fight against SARS-CoV-2 could exhibit a better prognosis.

Due to the remarkably different mRNA profiles between m^6^A cluster A and m^6^A cluster B, DEGs between the two clusters were labeled as m^6^A-DEGs, which were tightly associated with m^6^A modification. Consistent with the m^6^A classification, two genomic subtypes were identified by m^6^A-DEGs based on the unsupervised classification. Moreover, patients in m^6^A cluster B were more likely to be admitted to the ICU than m^6^A cluster A patients. Considering the individual heterogeneity of the immune system, it is necessary to establish an evaluation signature to reflect the individual m^6^A pattern. Here, based on m^6^A-DEGs, we defined an “m^6^A score” to quantify the m^6^A pattern for each COVID-19 individual. Patients in m^6^A cluster A presented higher HFD45, which meant that they had a better prognosis. In addition, similar to previous results, the m^6^A score was positively correlated with glycan metabolism, highlighting the core role of the m6A score in glucose metabolism. Furthermore, the clinical value of the m6A score was evaluated. Patients who were not admitted to ICU, did not have diabetes, or had not been treated by mechanical ventilation presented a relatively low median m6A score. These results further confirmed that the m^6^A score could serve as a satisfactory prognostic indicator.

Finally, we constructed a protective model with nine identified genes (*CHEK1*, *NDC80*, *PBK*, *H2BC11*, *TMSB4X*, *RPH3A*, *TM9SF1*, *EEF1D*, and *SNAPC2*) to predict patients who had COVID-19. Coincidently, some of the genes are linked to viruses infecting humans. *CHEK1*, which is a gene that is necessary for responding to DNA damage, was reported to be a potential target of saikosaponins which might function as an adjuvant therapy for COVID-19 patients (43). Bioinformatics analysis revealed that NDC80 and PBK can serve as biomarkers for HBV-associated hepatocellular carcinoma (44). Studies have reported that H2BC11 is associated with interferon signaling during viral infections (45). EEF1D, which serves as a guanine nucleotide exchange factor, can inhibit the nuclear import of the nucleoprotein and PA-PB1 heterodimer of the influenza A virus (46). Additionally, some of the genes are essential for immune system activation. For instance, RPH3A is known to be important for neutrophil integrin activation and TM9SF4 is required for cellular immunity in *Drosophila* (45, 47). The enrichment analysis revealed that external stimulation, cell cycle, and viral carcinogenesis might be the mechanisms underlying this protective model.

The model achieved a high AUC value in the training and validation sets. More importantly, patients without COVID-19 displayed higher protective scores compared to patients with COVID-19. In addition, previous studies have reported that patients with severe COVID-19 had relatively high crp and higher SOFA and APACHE-II scores (48–50). Consistent with the above findings, the results of the correlation analysis suggested that protective score was negatively correlated with the crp, SOFA score, and APACHE-II score. At the same time, protective score was positively correlated with HFD45 and ventilator-free days, both of which are indicators of favorable outcomes. These findings demonstrate that the protective score is an excellent indicator of clinical outcomes and prognosis in COVID-19 patients.

One limitation of our study was the lack of additional clinical confirmation for the expression levels of m^6^A-related genes and performance of the protective model. Furthermore, due to the vague survival information provided in the GSE157103 dataset, we could not analyze the precise prognostic value for the m^6^A score and protective model. Nevertheless, HFD45 can reflect a rough prognostic condition to some extent.

In conclusion, this study revealed the correlation between m^6^A regulators and lymphocytes and discovered the discrepant immune infiltration characteristics among COVID-19 patients with different m^6^A modifications. The m^6^A scoring system can effectively predict the clinical outcomes of patients with COVID-19. Importantly, the protective model based on nine signatures was capable of accurately identifying patients with COVID-19. In summary, our work provided novel insights into m^6^A modification in blood lymphocytes of patients infected with SARS-CoV-2 and an evaluation system to predict the clinical prognosis and possibility of contracting the COVID-19. Based on these findings, m^6^A DEGs can serve as biomarkers to detect suspected or confirmed SARS-CoV-2 carriers; however, further research is required to uncover the mechanism underlying elevated expression of m^6^A methylation modification in the lymphocytes of infected individuals.

## Data Availability Statement

Publicly available datasets were analyzed in this study. These data can be found here: National Center for Biotechnology Information (NCBI), Gene Expression Omnibus (GEO), https://www.ncbi.nlm.nih.gov/geo/, GSE157103.

## Author Contributions

JC designed and supervised the study, XQ collected the data, performed all data analysis and drafted the manuscript. XQ and JC performed original draft preparation, writing, review and editing. XH provided analytical technical support. QL and QZ were responsible for the data acquisition and critical reading of the manuscript. All authors reviewed and approved the final version of the manuscript.

## Funding

This study was supported by the Science and Technology Research Plan Project of Chongqing Education Commission (KJQN202100418), the Natural Science Foundation of Chongqing (No. cstc2021jcyj-msxm0317) and the National Natural Science Foundation of China (No. 82030065).

## Conflict of Interest

The authors declare that the research was conducted in the absence of any commercial or financial relationships that could be construed as a potential conflict of interest.

## Publisher’s Note

All claims expressed in this article are solely those of the authors and do not necessarily represent those of their affiliated organizations, or those of the publisher, the editors and the reviewers. Any product that may be evaluated in this article, or claim that may be made by its manufacturer, is not guaranteed or endorsed by the publisher.
