# Supplementary material for: m6A Regulator-Mediated Methylation Modification Patterns and Characteristics of Immunity in Blood Leukocytes of COVID-19 Patients
Source: Front Immunol. 2021 Nov 30;12:774776. doi: 10.3389/fimmu.2021.774776 (PMC8669770; doi:10.3389/fimmu.2021.774776)
Supplement: Supplementary file 1 [file DataSheet_1.pdf]

Figure S1 (A-C) Consensus clustering matrix based on 20 m^6A genes for k=3-5. **(D-E)** The consensus distribution and Delta area for k=2-5. **(F)** The PCA of the m^6A clusters.

Figure S2 (A-C) The previral genes between the m^6A clusters

Figure S3 (A-C) The stimular genes **(A)**, inhibitor genes **(B)** and MHC genes **(C)** between the m^6A clusters. *p<0.05, **p<0.01, ***p<0.001, ****p<0.0001.

Figure S4 (A-C) Consensus clustering matrix based on 6771 DEGs for k=2-5. **(D)** The consensus distribution area for k=2-5. **(E)** The PCA of the Gene clusters. **(F)** The 20 m^6A genes between the Gene clusters. *p<0.05, **p<0.01, ***p<0.001, ****p<0.0001.

Figure S5 (A-B) The HFD45 **(A)** and m6A score **(B)** between the Gene clusters. **(C-F)** The boxplot of m6A score in groups of different clinical characteristics including sex **(C)**, ICU **(D)**, mechanical ventilation **(E)** and diabetes **(F)**. **(G)** The correlation between m6A score and common pathways.

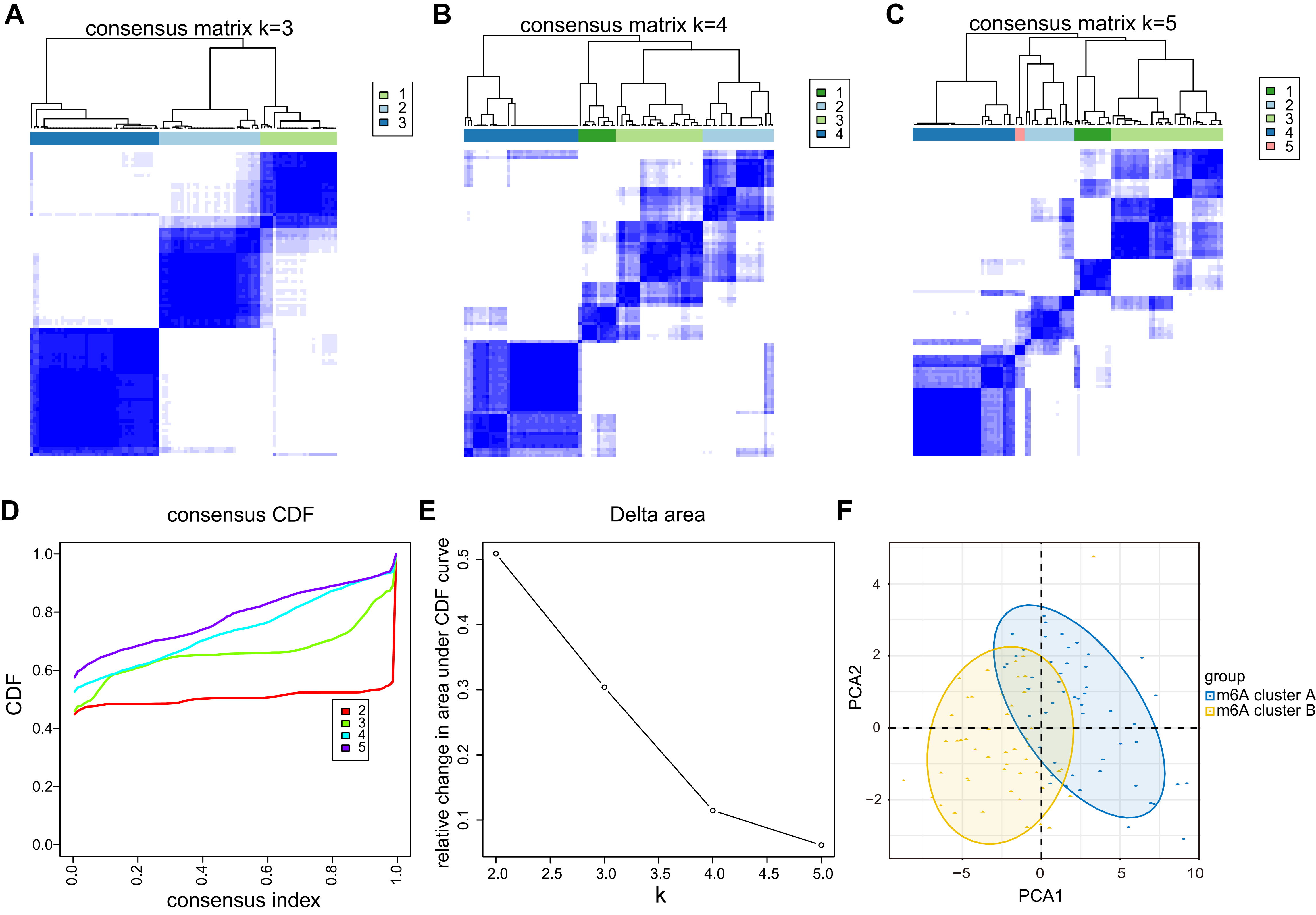
A
consensus matrix k=3
1
2
3
B
consensus matrix k=4
1
2
3
4
C
consensus matrix k=5
1
2
3
4
5
D
consensus CDF
CDF
consensus index
1.0
0.8
0.6
0.4
0.2
0.0
2
3
4
5
E
Delta area
relative change in area under CDF curve
k
2.0
2.5
3.0
3.5
4.0
4.5
5.0
F
PCA2
PCA1
group
m6A cluster A
m6A cluster B
−5
0
5
10
4
2
−2

Figure S1

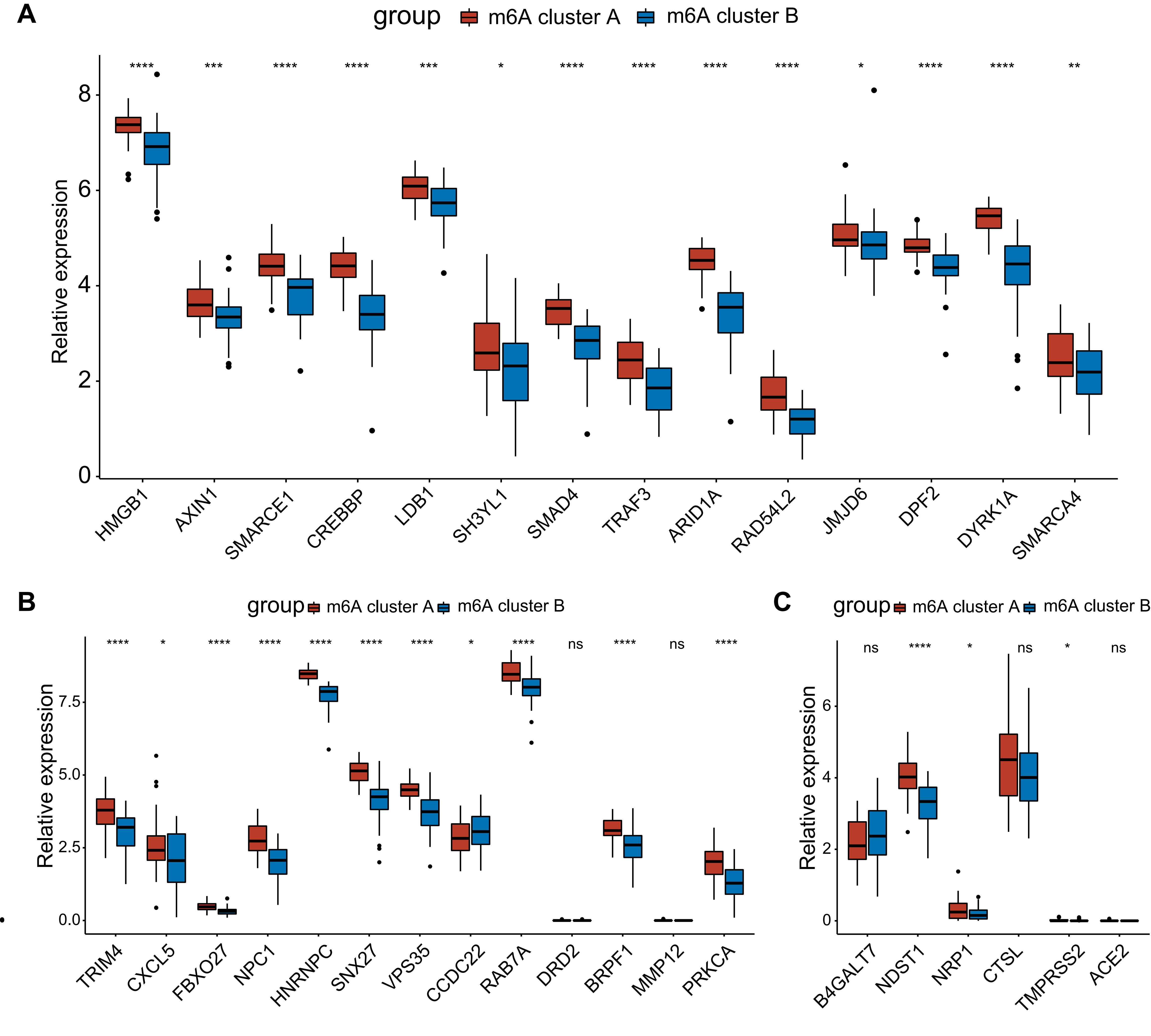
A
group m6A cluster A m6A cluster B
Relative expression
**** *** **** **** *** * **** **** **** **** * **** **** **
HMGB1
AXIN1
SMARCE1
CREBBP
LDB1
SH3YL1
SMAD4
TRAF3
ARID1A
RAD54L2
JMJD6
DPF2
DYRK1A
SMARCA4
B
group m6A cluster A m6A cluster B
Relative expression
**** * **** **** **** **** **** * **** ns **** ns ****
TRIM4
CXCL5
FBXO27
NPC1
HNRNPC
SNX27
VPS35
CCDC22
RAB7A
DRD2
BRPF1
MMP12
PRKCA
C
group m6A cluster A m6A cluster B
Relative expression
ns **** * ns * ns
B4GALT7
NDST1
NRP1
CTSL
TMPRSS2
ACE2

Figure S2

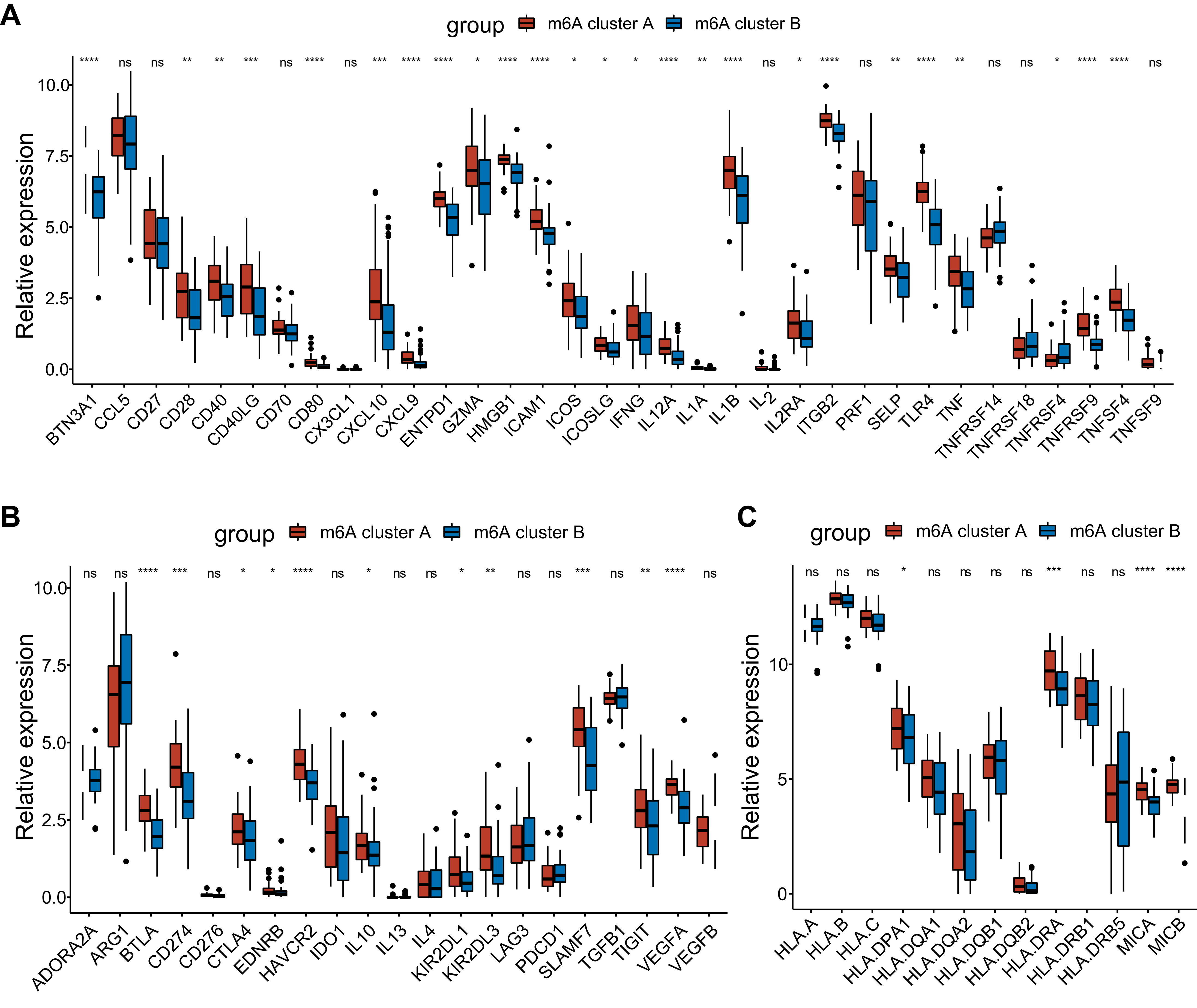

A
group m6A cluster A m6A cluster B
Relative expression
BTN3A1 CCL5 CD27 CD28 CD40 CD40LG CD70 CD80 CX3CL1 CXCL10 CXCL9 ENTPD1 GZMA HMGB1 ICAM1 ICOS ICOSLG IFNG IL12A IL1A IL1B IL2 IL2RA ITGB2 PRF1 SELP TLR4 TNF TNFRSF14 TNFRSF18 TNFRSF4 TNFRSF9 TNFSF4 TNFSF9
B
group m6A cluster A m6A cluster B
Relative expression
ADORA2A ARG1 BTLA CD274 CD276 CTLA4 EDNRB HAVCR2 IDO1 IL10 IL13 IL4 KIR2DL1 KIR2DL3 LAG3 PDCD1 SLAMF7 TGFB1 TIGIT VEGFA VEGFB
C
group m6A cluster A m6A cluster B
Relative expression
HLA.A HLA.B HLA.C HLA.DPA1 HLA.DQA1 HLA.DQA2 HLA.DQB1 HLA.DQB2 HLA.DRA HLA.DRB1 HLA.DRB5 MICA MICB

Figure S3

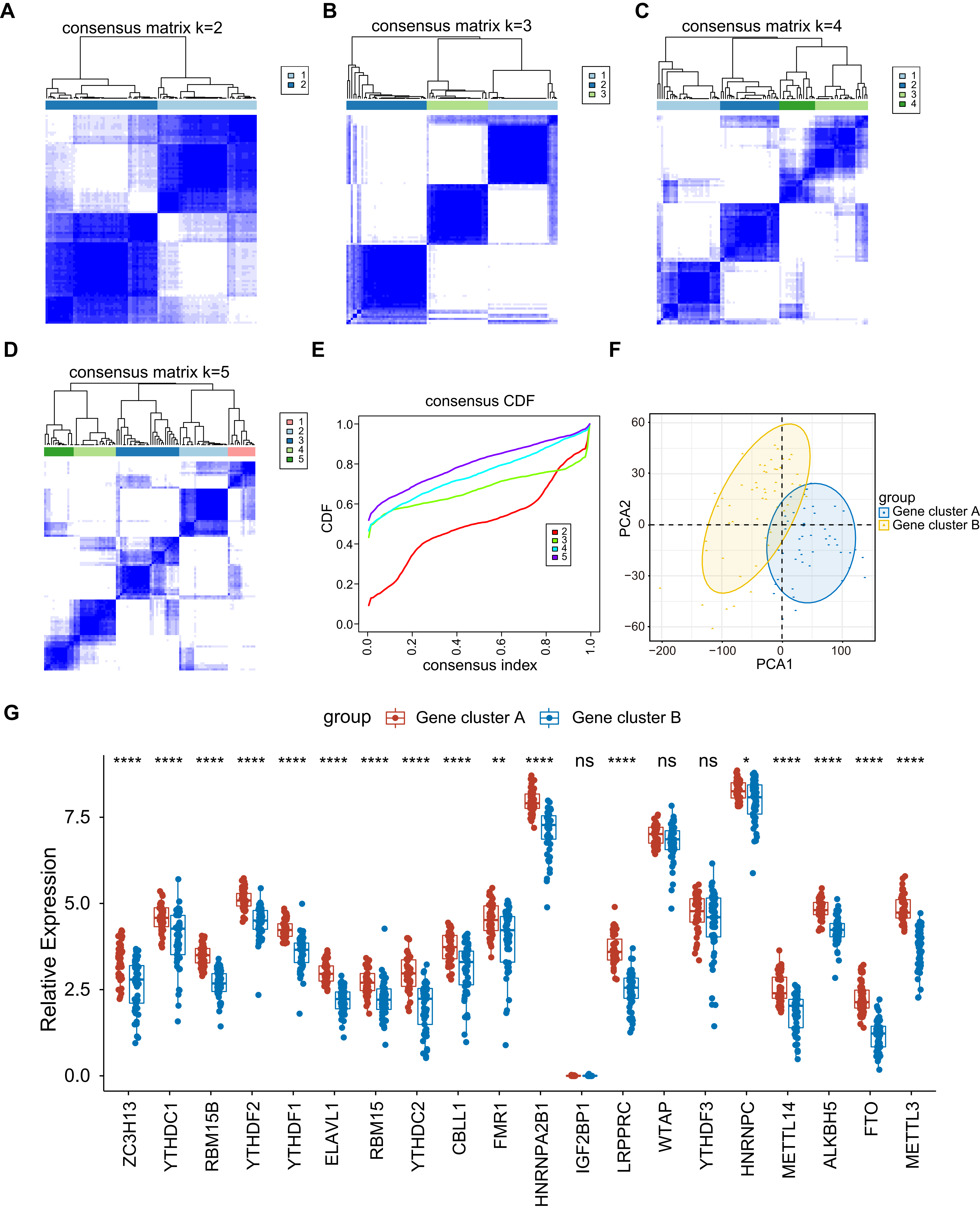
A
consensus matrix k=2
B
consensus matrix k=3
C
consensus matrix k=4
D
consensus matrix k=5
E
consensus CDF
CDF
consensus index
F
PCA1
PCA2
group
Gene cluster A
Gene cluster B
G
group
Gene cluster A
Gene cluster B
Relative Expression
**** **** **** **** **** **** **** **** **** ** **** ns **** ns ns * **** **** **** ****
ZC3H13
YTHDC1
RBM15B
YTHDF2
YTHDF1
ELAVL1
RBM15
YTHDC2
CBLL1
FMR1
HNRNPA2B1
IGF2BP1
LRPPRC
WTAP
YTHDF3
HNRNPC
METTL14
ALKBH5
FTO
METTL3

Figure S4

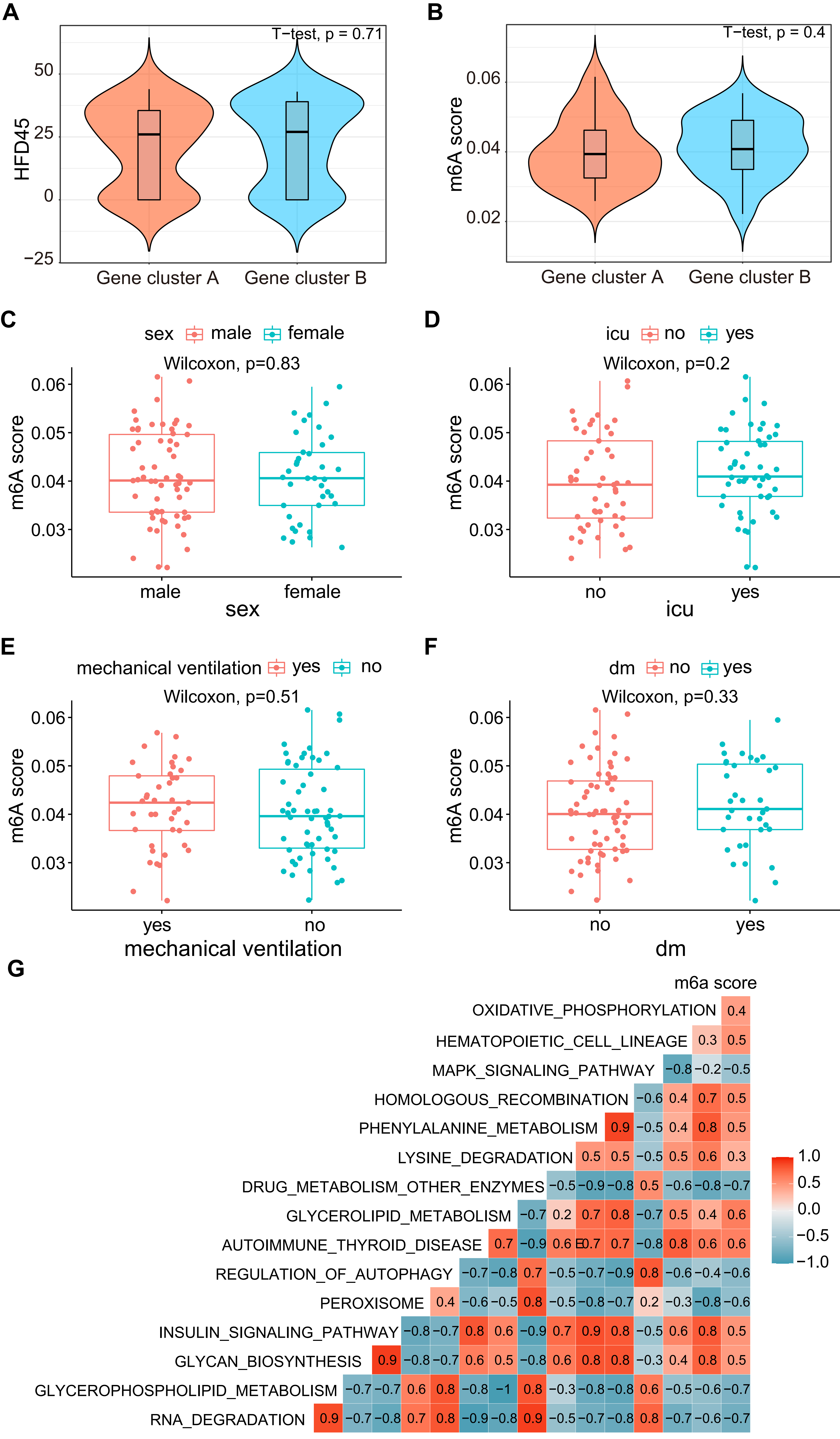
A
T−test, p = 0.71
HFD45
50
25
0
−25
Gene cluster A
Gene cluster B
B
T−test, p = 0.4
m6A score
0.06
0.04
0.02
Gene cluster A
Gene cluster B
C
sex
male
female
Wilcoxon, p=0.83
m6A score
0.06
0.05
0.04
0.03
male
female
sex
D
icu
no
yes
Wilcoxon, p=0.2
m6A score
0.06
0.05
0.04
0.03
no
yes
icu
E
mechanical ventilation
yes
no
Wilcoxon, p=0.51
m6A score
0.06
0.05
0.04
0.03
yes
no
mechanical ventilation
F
dm
no
yes
Wilcoxon, p=0.33
m6A score
0.06
0.05
0.04
0.03
no
yes
dm
G
m6a score
OXIDATIVE_PHOSPHORYLATION
HEMATOPOIETIC_CELL_LINEAGE
MAPK_SIGNALING_PATHWAY
HOMOLOGOUS_RECOMBINATION
PHENYLALANINE_METABOLISM
LYSINE_DEGRADATION
DRUG_METABOLISM_OTHER_ENZYMES
GLYCEROLIPID_METABOLISM
AUTOIMMUNE_THYROID_DISEASE
REGULATION_OF_AUTOPHAGY
PEROXISOME
INSULIN_SIGNALING_PATHWAY
GLYCAN_BIOSYNTHESIS
GLYCEROPHOSPHOLIPID_METABOLISM
RNA_DEGRADATION
1.0
0.5
0.0
−0.5
−1.0

Figure S5
